# The digital cumulative complexity model: a framework for improving engagement in digital mental health interventions

**DOI:** 10.3389/fpsyt.2024.1382726

**Published:** 2024-09-03

**Authors:** Shane P. Cross, Mario Alvarez-Jimenez

**Affiliations:** ^1^ Orygen Digital, Orygen, Parkville, Melbourne, VIC, Australia; ^2^ Centre for Youth Mental Health, University of Melbourne, Melbourne, VIC, Australia

**Keywords:** digital mental health, engagement, treatment burden, patient capacity, minimally disruptive medicine, cumulative complexity model, person-centered care

## Abstract

Mental health disorders affect a substantial portion of the global population. Despite preferences for psychotherapy, access remains limited due to various barriers. Digital mental health interventions (DMHIs) have emerged to increase accessibility, yet engagement and treatment completion rates are concerning. Evidence across healthcare where some degree of self-management is required show that treatment engagement is negatively influenced by contextual complexity. This article examines the non-random factors influencing patient engagement in digital and face-to-face psychological therapies. It reviews established models and introduces an adapted version of the Cumulative Complexity Model (CuCoM) as a framework for understanding engagement in the context of digital mental health. Theoretical models like the Fogg Behavior Model, Persuasive System Design, Self-Determination Theory, and Supportive Accountability aim to explain disengagement. However, none adequately consider these broader contextual factors and their complex interactions with personal characteristics, intervention requirements and technology features. We expand on these models by proposing an application of CuCoM’s application in mental health and digital contexts (known as DiCuCoM), focusing on the interplay between patient burden, personal capacity, and treatment demands. Standardized DMHIs often fail to consider individual variations in burden and capacity, leading to engagement variation. DiCuCoM highlights the need for balancing patient workload with capacity to improve engagement. Factors such as life demands, burden of treatment, and personal capacity are examined for their influence on treatment adherence. The article proposes a person-centered approach to treatment, informed by models like CuCoM and Minimally Disruptive Medicine, emphasizing the need for mental healthcare systems to acknowledge and address the unique burdens and capacities of individuals. Strategies for enhancing engagement include assessing personal capacity, reducing treatment burden, and utilizing technology to predict and respond to disengagement. New interventions informed by such models could lead to better engagement and ultimately better outcomes.

## 1 Introduction

Mental ill health adversely affects significant numbers of people worldwide (1), 1 in 5 in any given year (2). Recent data from Australia indicates that 21.5% of the population and 38.8% of young people aged 16-24 years experienced a mental disorder in the previous 12-months. Of these people, 45.1% saw a health professional, 21.3% saw a psychologist and 4.8% accessed a digital mental health service (3). Biological and psychological treatments form the main basis for mental health treatment, but their coverage and ability to substantially reduce disease burden is low (4). While the majority of patients prefer psychotherapy over medication (5), access to these treatments is restrictive due to cost, distance and availability of trained therapists (4). The advent of the internet has helped improve access to psychological treatments (6), overcoming barriers to traditional service provision such as distance, availability, and stigma (7).

Despite these advances, sustained engagement and completion of digital psychological therapy remains a challenge for many patients, where on average, 38.7% do not complete the assigned treatment (8). In comparison, a large scale meta-analysis of engagement in face-to-face settings showed disengagement or dropout rates vary from 17.8% to 37.6% (9). A challenge for the field, however, has been the various definitions and measurement of engagement in both digital or remote settings (10), and face-to-face settings (9). In the digital context, engagement has been defined variously. Within DMHI, engagement is primarily defined by user behaviors such as uptake, sustained use and adherence to the intervention at the intended frequency or duration (11). More specific definitions include the users level of attention, interest and their temporal, emotional and cognitive investment, and include a range of broad actions, including logging in or reading therapeutic content, responding to notifications, and engaging in off-line behavioral change such as behavioral activation (12, 13).

Notwithstanding precise definitions, treatment engagement is critical, as it has also been shown to be a vital mediator and moderator of treatment particularly the quality of that engagement as it relates to the use of specific therapeutic activities that translate to clinical outcomes (14). While engagement with DMHI mediates outcome, various factors have also been shown be associated with engagement itself. Regarding patient or user characteristics, there appear to be common factors associated with disengagement that apply to both digital and face-to-face formats, including lower age, male gender, lower educational status, lower therapist support, among others (9, 15–17). Characteristics associated with engagement are most often reported cross-sectionally at the group level, obscuring the complex and dynamic interactions between these variables over time. Furthermore, studies vary in the number of contextual factors they measure, and rarely capture in the one study the full range of factors known to be associated with psychological treatment disengagement.

While low engagement and engagement variability in both digital and face-to-face mental health is well documented, theories or specific mechanisms that drive this variability has generally been under-researched. In the digital context, various models and theories have been put forward to explain engagement. Some of the prominent models and theories are outlined below.

### 1.1 The Fogg behavior model and persuasive system design

The Fogg Behavior Model (FBM) provides a structured framework for understanding human behavior in the context of technology use and digital engagement (18). The model aims to support behavior change through persuasive design, and asserts that behavior is a product of three factors: motivation, ability, and triggers, and that for a person to perform a target behavior, they must be sufficiently motivated, have the ability to perform the behavior, and be triggered to perform the behavior (18). This model is particularly useful in the design of digital health technologies, where engaging users effectively requires understanding and influencing their motivations, enhancing their ability to use the technology often by making the technology easy to use, and providing timely and appropriate prompts or reminders. Persuasive system design (PSD) builds upon FBM to describe technology design techniques that can motivate behavior change and user engagement, comprising of 28 persuasive strategies grouped into four categories—primary task support, dialogue support, system credibility support, and social support (19). The evidence on how effective these approached are in improving engagement is limited. In one recent review, a negative association between PSD features and engagement (as measured by completion rate) was observed (20). Another found mixed evidence that a specific strategy or group of strategies is relatively more efficacious in promoting engagement than others (21).

### 1.2 Self determination theory

Self Determination Theory (SDT) is one of the most prominent and empirically supported theories of human motivation that has demonstrated efficacy in predicting motivated behavior in multiple contexts and populations and for a variety of health behaviors such as physical activity, healthy eating, and smoking cessation (22). The quality of motivation is influenced by the extent to which individuals experience support for three basic psychological needs: autonomy, competence, and relatedness. Therefore, behaviors or messages from agents that support the satisfaction of these needs are likely to promote autonomous motivation and sustained behavior change within the individual, while those that do not may undermine autonomous motivation and lead to negative outcomes, such as disengagement or poor adherence.

### 1.3 Supportive accountability

In acknowledging the value of support in improving engagement and adherence with psychological interventions, Mohr and colleagues constructed the Supportive Accountability (SA) model (23), which is particularly relevant in the context of remote interventions where there is limited direct human interaction. Support emphasizes the encouragement, guidance and feedback from a trained coach or therapist. Accountability refers to the user justifying their actions or inactions to another person such as the coach or therapist. Both SDT and SA emphasize the importance of interpersonal interactions and relationships on motivation and engagement.

The above theories and models are widely used and referenced when developing digital interventions, though each when used on its own has limitations when applied to the use of digital interventions to treat mental ill health. For example, in their extensive review of engagement with DMHI, Borghouts and colleagues (17) coded 3 categories of barriers and facilitators: 1. user characteristics, including demographics such as age and education, personality traits, mental health status and severity, beliefs, technology experience and skills and life integration, 2. program characteristics including type of content, perceived fit, perceived usefulness, level of guidance or support, social connectedness, perceived impact or effectiveness of the intervention, and 3. technology and environment characteristics which include technology related factors, technical issues, usability and delivery platform, privacy and confidentiality, social influence by external others, and implementation factors. Based on this evidence, it’s clear that a more comprehensive and holistic models of digital psychological treatment engagement are required. Models that consider the complex interaction between personal capacity and the burdensomeness of the technologies and interventions themselves. For example, each of the above models do not adequately explain the complex interacting relationship between the personal resources of the patient/user, the burden of their illness, the burden of their current life circumstance and the burden placed upon them by the DMHI (including both the technology and the intervention itself). In the following section we outline an approach to conceptualizing treatment engagement and adherence used more widely in chronic disease research that has direct relevance for digital psychological interventions.

## 2 The cumulative complexity model

**Figure 1:**
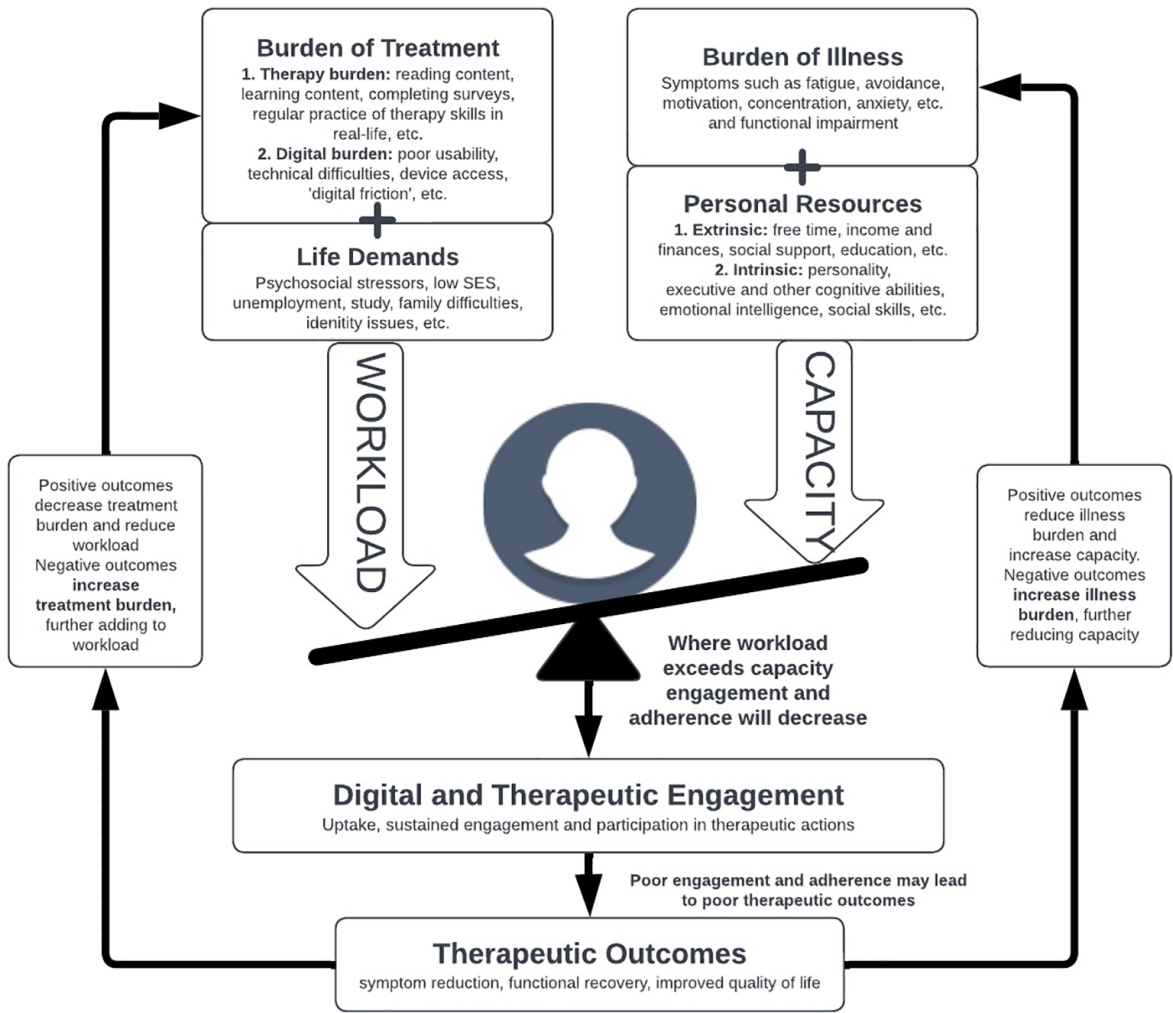
The digital cumulative complexity model (DiCuCoM) adapted for digital mental health interventions, highlighting the isolated and cumulative impact of workload and capacity as they interact over time.

The cumulative complexity model (CuCoM) (24) was developed in the context of multimorbidity and chronic conditions. It is a person-centered functional complexity model that emphasizes the interplay between a patient’s workload of demands (encompassing the burdens associated with doing the ‘work’ or treatment and the other contextual burdens operating in the person’s day-to-day life) and their capacity (comprising the capacity reducing impact of the illness itself as well as personal resource scarcity) to manage this workload (**Figure 1**). While excessive workload or low capacity in isolation may contribute to poor access or nonadherence to an intervention, CuCoM argues that it is the *imbalance* between the two which is the primary driver poor engagement and subsequent poor outcomes (24). Over time, especially if treatments are ineffective, the burden of illness (BOI) and the burden of treatment (BOT) act as feedback loops, further eroding capacity and adding additional workload and demands, resulting in a cumulative cycle of patient complexity (24). CuCoM has gained research attention and clinical application in relation to chronic disease management (25–29), but has yet to be applied in mental health or digital mental health contexts. The following section outlines the core components of an adapted version of the CuCoM, the Digital Cumulative Complexity Model (DiCuCoM) as they each relate to digital psychological interventions. **Table 1** outlines the key similarities and differences between DiCuCoM and other the other prominent theories and models of digital mental health user engagement outlined above.

**Table 1:** Similarities and difference of the DiCuCoM with prominent other theories of models of DMHI user engagement.

Model/Theory and Focus	Key Components	Application in DMHI	Similarity with DiCuCoM	Difference with DiCuCoM
**Cumulative Complexity Model** ***Balancing workload and patient capacity in healthcare settings***	Workload, Patient Capacity, Balancing Factors	Emphasizes managing workload and capacity for effective healthcare engagement	Same general framework.	Adds digital user requirements and context in addition to health behavior change requirements.
**Fogg Behavior Model (Fogg) and Persuasive System Design** ***Immediate behavior change through motivation, ability, and prompts***	Motivation, Ability, Prompts	Used for designing immediate, action-oriented digital health interventions	Recognizes the need to balance the complexity and burden of using a health intervention with the user’s capacity. This can lead to designs that are more user-friendly and more supportive (e.g., prompts).	Does not specifically address the balance of workload and personal capacity in the broader context of healthcare management, or how this balance can impact patient engagement over time.
**Self Determination Theory** ***Intrinsic motivation and psychological needs (autonomy, competence, relatedness)***	Autonomy, Competence, Relatedness	Focuses on long-term engagement and intrinsic motivation in digital health	Acknowledges the impact of individual capacities (known as competencies in SDT) and needs on behavior.	SDT more focused on individual’s internal psychological needs than external workload and the practical aspects of managing healthcare, specifically the balance and ongoing interaction between workload and personal capacity.
**Supportive Accountability** ***Adherence through external support and accountability***	Human Support, Accountability	Enhances adherence to digital health interventions through support and accountability	Both models emphasize the importance of external support. In the Cumulative Complexity Model, support can be a factor that increases a person’s capacity to manage their health workload.	SA centers on accountability and support, while CuCoM also acknowledges the individuals’ differences in broader burdens (treatment induced and life demands) and resources (personal capacity and illness burden) to personalize the level of support required.

### 2.1 Workload or burden and DMHI

Being a patient can be hard work (30–32). In addition to the burden of illness (BOI) experienced by the individual (expanded on below), individuals may experience concurrent burdens in the ordinary demands of daily living (life demands). When seeking treatment for a mental health problem, there is additional burden or work placed on the patient (or a user of a digital intervention). This is known as the Burden of Treatment (BOT). Both impact complexity through their toll on the persons time, effort, and attention (24).

#### 2.1.1 Life demands

There is evidence that highlights the relationship between high burden life demands and engagement in DMHI (33). Work commitments, family problems, moving house (34), financial concerns or ‘life chaos’ (defined as numerous commitments or unstable living arrangements) (35), being unemployed (36), and experiencing a greater number of these psychosocial stressors in daily life (16) are all associated with reduced engagement.

#### 2.1.2 Burden of treatment

In addition to whatever burdens an individual experiences in their daily life and from their illness, DMHI themselves require work and therefore carry subjective burden. Compared to face-to-face treatments, DMHI have been highly successful at reducing BOT, saving time and financial resources to physically attend appointments for example. However other treatment burdens remain, both in navigating the technology itself (digital burden) and the work of engaging in cognitive and behavioral change (therapy burden). Examples include reading lengthy and cognitively demanding content (33), practicing cognitive or behavioral skills that trigger negative emotional states (33), completing numerous or lengthy questionnaires, setting time aside to practice skills (therapy burden) (37); and experiencing technical issues (38), poor usability (37), difficulty logging on, difficulty navigating the platform, content that is delivered in non-preferred formats (audio-visual versus text alone), content that is difficult to read (digital burden) (17, 39, 40).

### 2.2 Personal capacity and DMHI

Capacity can be thought of as the sum-total of resources and abilities that a patient can draw on to access care, use care, and enact self-care. Every structure or system (including patients) has a maximum performable workload that is determined by its unique capacity (32). Capacity is affected by the availability or scarcity of personal resources, be they intrinsic (such as physical, mental, personal attitudes and beliefs, personality, intelligence, literacy) or extrinsic (such as socio-economic resources, social supports, education level, income, amount of free time). Furthermore, capacity is negatively affected by the burdens associated with their disease or illness itself (BOI). The interaction between personal resources and BOI affect the person’s ability or readiness to do the ‘work’ of treatment (24). Importantly, capacity varies between individuals; some persevere despite tremendous workloads; others falter even when relatively unencumbered (24). According to Shippe et al. (24), measurable attributes of capacity include the following: 1. amount/magnitude (e.g., greater/lesser symptoms, finances, or social support); 2. controllability (some factors, such as literacy, are more responsive to personal efforts than others, such as pain); 3. extensiveness or scope of impact (symptoms may have limited or widespread effects on functioning).

#### 2.2.1 Personal resources

In the context of DMHI, personal capacity has consistently been directly or indirectly implicated in engagement. Indirectly, personal or demographic factors such as younger age (16, 41), being male (42–44) (17, 39), lower education level (15, 17). These factors have an indirect effect, partly because factors such as age are correlates to other abilities that affect capacity, such as underdeveloped executive functioning skills such as self-monitoring and organization (45), and increased disease and lifestyle risk factor burdens that accompany adolescence (46, 47). Direct factors include possessing conscientious personality traits (48, 49), possessing negative personal beliefs about treatment including stigma (35) (17, 50), possessing low rates of mental health literacy (17, 51); having little free time (17, 52). Socioeconomic status may also be an indirect indicator of capacity as it is defined as a rough measure of the relative material resources or nonmaterial resources a person may have, including education, occupational prestige, and neighborhood quality (53). As a correlate of reduced capacity, SES has been shown to be associated with improved physical health and lower rates of heart disease, stroke, cancer, diabetes, and many other serious illnesses as well as lifespan (54). Mental health also increases with SES, with progressively less depression, anxiety, and psychosis at higher levels of SES (55–57).

#### 2.2.2 Burden of illness

Burden of illness (BOI) factors associated with reduced DMHI engagement include higher symptom severity or complexity (16, 17, 41, 43), physical illness (52), and the lack of motivation inherent in conditions such as depression (52). The stage or severity of illness in young adults (58, 59) has also been shown to negatively affect treatment engagement in face-to-face services, especially in those with greater symptom severity, functional impairment and ambiguous syndromes (60, 61). While not specifically examined to date, it is possible that different symptoms or symptom combinations may also have distinctive negative effects on personal capacity and subsequently treatment engagement. For example, the symptom of amotivation may reduce the capacity of an individual to do behavioral activation tasks, hyperactivity may make it difficult to do mindfulness activities, rigid and fixed beliefs may make cognitive challenging difficult and so on.

### 2.3 Workload/burden and capacity interactions

The core assertion of the model is that where there is an imbalance between the person’s workload and their capacity to meet those demands, poor engagement and subsequently poor outcomes ensue (24). Collectively, a person’s capacity may or may not reach that required to meet the prerequisites of sustained engagement in any psychological treatment. When workload exceeds capacity, people feel the effects of treatment burden and this can lead to de-prioritization of various aspects of care and ultimately result in poor fidelity to treatment programs and to treatment failures (28, 30, 62–64). An example is reading content in a DMHI. The act of reading is considered work and people vary in their capacity to complete this work. Those with high reading ability will not experience this work as burdensome. Those with reading difficulties will find that the reading workload exceeds their reading capacity and will find this burdensome, leading to disengagement. Furthermore, people experiencing this imbalance may disengage or clinically deteriorate, leading service providers to provide more care, often unintentionally fueling burden and creating a perpetuating cycle of increasing complexity. Approaches like stepped care models or adaptive interventions increase the intensity and nature of treatment in response poor engagement or poor treatment outcome (65) but in doing so, may increase the burden of treatment in a potentially already ‘overloaded’ person. This is why most stepped care models recommend greater levels of professional support as a means to increase capacity in the face of increase illness and treatment burden (65).

Given these complexities, multiple interactions may shape patient demands, capacity, and the interplay between them (**Figure 1**). Even though there is significant evidence of both burden and capacity related factors in isolation influencing engagement, limited research has been conducted on these complex interactions over time. Yet it is their interaction at the individual level which is most likely to be predictive of engagement and subsequent outcome, which may explain the variability in findings when these factors are studied in isolation. The goal is to balance the recommended treatment workload with the unique capacity of the individual to avoid overload and to adapt this balance over the course of care. Engagement research, however, rarely examines how these complex combinations interact, which inhibits our ability to design, test and deliver more appropriate interventions.

The workload-capacity imbalance is most apparent in standardized DMHI (and other psychological treatments for that matter), where all users receive the same intervention regardless of their capacity. While useful for research standardization and fidelity, these ‘one-size-fits-all’ approaches disregard individual variation in illness burden and personal resources. Treatment demands are fixed and usually specify a set amount of time to spend, a set amount to read, what tasks to complete, and the frequency and length of support appointments to participate in. The result as outlined above is poor engagement for large numbers of participants in these standardized programs.

## 3 Addressing workload-capacity imbalances in psychological interventions

Developed alongside the CuCoM, the Minimally Disruptive Medicine (MDM) model (30) advocates for an approach to healthcare that seeks to tailor treatment to the realities of patients’ lives, minimizing the burden of treatment while maximizing patient capacity. The core principle of MDM is to carefully calibrate the treatment and broader healthcare workload imposed on patients, ensuring it does not overwhelm their capacity. MDM aims to achieve a balance by considering the demands of treatment in the context of the patient’s life and responsibilities, striving for the best clinical outcomes with the least possible impact on a patient’s daily functioning and quality of life (66). This model is person-centered, recognizing the importance of patient goals and preferences, and seeking to avoid the treatment or health system-related exacerbation of health issues due to overwhelming intervention and management regimens (32). For example, self-managing a chronic disease is estimated to demand two hours of a patient’s attention and effort per day (67).

**Figure 2:**
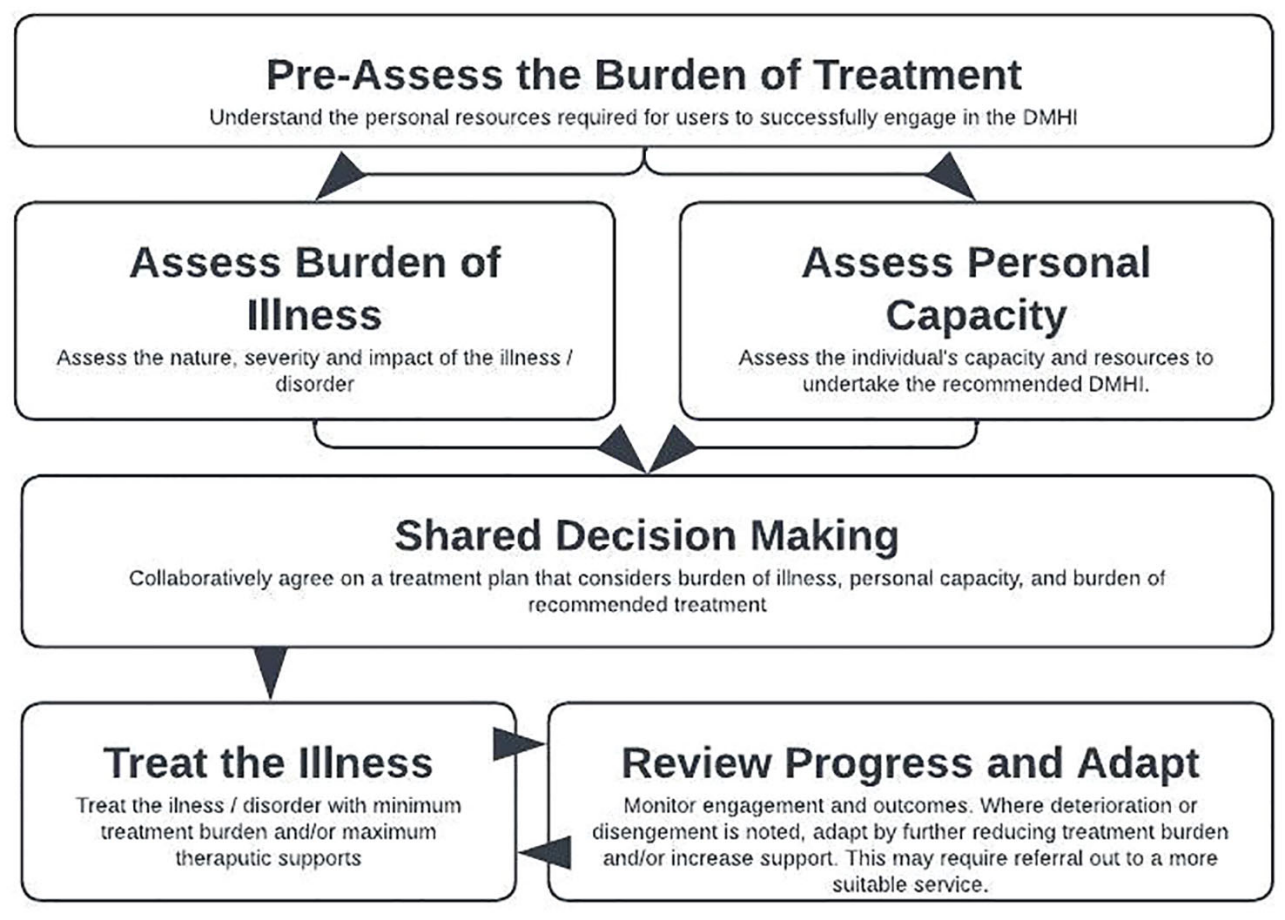
A model for addressing patient complexity to facilitate sustained engagement in psychological interventions.

There is an urgent need to develop new psychological treatments and models to minimize burden and ensure engagement in mental health interventions. Models that are more nuanced, context-sensitive, and responsive to individual variation in capacities and burdens. This approach aligns with the ethos of MDM by ensuring that interventions, be they face-to-face or digital, do not inadvertently exacerbate disparities by imposing a one-size-fits-all treatment regime that ignores the complexities of patients’ lives. **Figure 2** outlines a person (user)-centered approach to providing psychological interventions to patients that considers burden, capacity in addition to traditional disease/problem assessment and intervention.

## 4 Strategies to enhance engagement in DMHIs using the DiCuCoM

Next, we outline two strategies that can be used to provide and develop more personalized psychological interventions derived from the DiCuCoM.

### 4.1 Utilize shared decision making to determine the appropriateness of the DMHI

Shared decision making and informed consent are critical before commencing any health intervention (68) following an adequately comprehensive assessment of the user at entry (**Figure 2**). Users should have a complete understanding of the potential benefits, burdens, demands, harms and risks of starting a proposed intervention. These kinds of discussions with MHPs are valued by users of DMHI (69). Indeed, a recent meta-analysis showed that synchronous contact before the digital intervention commenced significantly improved intervention adherence and outcome (70). For example, if the intervention requires an 8-week enrolment, weekly reading, regularly questionnaire completion and regular telephone contact with the clinician, these requirements should be stated clearly up front, and the user themselves will decide as to their capacity to engage in the suggested work to obtain their desired goal. Just like other mental health interventions, DMHIs are not suitable for all. It is important that the intended user’s assessed capacity is balanced against the fixed burden of the intended intervention, and that this is openly and transparently discussed and consented to prior to commencement. If this were to occur, less users might start these interventions, but conceivably more would engage. At present, we have many users who fail to complete an intervention, which either maintains or exacerbates their burden of illness, and can lead to ‘failure demand’, where failing to adequately treat the condition the first time creates later downstream demand on services (64, 71).

### 4.2 Provide additional supports to increase personal capacity

Providing additional professional support has been associated with improved engagement (39). A meta-analysis of 40 studies with a total of 7313 participants found an overall completion rate of 43%, with completion rates increasing with greater levels of support: 26% without support, 62% administrative support only, and 72% for therapeutic support (40). Studies have also explored the role of social support systems in psychological treatments, including peer support groups, community-based programs, and social networking (72, 73). These interventions aim to provide emotional and practical support, reducing feelings of isolation, shape more positive attitudes toward intervention and provide additional encouragement or motivation to do the work of therapy (74). Providing additional social supports may also be warranted, such as access to career consultants or services (75), information or direction to services that can assist with financial counselling, domestic violence, housing and accommodation. Digital Navigators are becoming increasingly popular as a way to support MHP and users with digital literacy, workflow integration (for clinicians) and engagement, although further work is required to standardize the scope of these roles (76).

## 5 Future research and development of DMHIs

As outlined, standardized DMHI have an inequitable effect on user engagement, simply because there is significant variability in the degree of contextual burden and internal capacity across users. To increase DMHI engagement and subsequent outcome, future research should explore ways of reducing treatment burden and offering supportive technologies that make treatment easier to access and complete. In a recent systematic review, young people reported that interventions are more likely to be used if they are low effort, fun, relaxing, easy to navigate, and fit into daily routine (39).

‘Effort-Optimized Interventions’ have been recommended as a way to reduce the effort required to engage in therapeutic activities, among other things, such as setting graded tasks and setting dynamically tailored tasks (77). Further, they argue that commerce and social media sectors use A/B testing paradigms (randomized controlled experiments where two versions of a variable such as a feature are compared) to evaluate small platform changes that lead to large improvements in engagement, and that similar approaches can be used in reducing effort in DMHIs (77). In this way creating a wider range of interventions that vary by effort, burden, or difficulty, and testing them for engagement and effectiveness, will provide users with more choice in selecting interventions that fit them in the context of their personal capacities. While developing these new interventions, users can rate and provide feedback on the level of difficulty or workload experienced for the whole DMHI, over and above technology usability alone. Researchers could identify gaps in their existing interventions and develop new ways of delivering them that carry less treatment burden. Recent examples include reducing word counts in iCBT programs (78), and single-module, ultra-brief versions of much longer programs (79), both proved to be as effective as their longer-form counterparts. ‘Just in Time Adaptive Interventions’ are another example of a new DMHI delivery technique that requires little user effort, and have been shown to be engaging and effective (80). Maintaining effectiveness while reducing effort is crucial. Knowing which are the core components that absolutely must be retained will be of critical research focus. Precision psychological interventions (81, 82) aim to differentiate the essential and non-essential components of treatment and pare back interventions to the core components when there is imbalance, which may result highly personalized interventions.

Research into the development of enhanced measurement-based care and routine outcome monitoring that captures metrics beyond symptoms (including capacity and burden metrics) is another avenue through which engagement might be improved. Disengagement is often determined in retrospect, after a DMHI was due to be completed. We do not yet have systems that can predict disengagement in near or real time, which reduces our ability to respond and intervene early to avoid treatment failure. Regularly measuring and feeding-back user progress to clinicians and users themselves, might ensure treatments are more efficiently targeted, potentially reducing unnecessary intervention, and tailoring the therapy more closely to user needs. Further research using machine learning or artificial intelligence might explore a broader range of burden, capacity and maintenance factors and the nature of their relationship with uptake, engagement, and outcome, which may inspire the development of better targeted, more precise, and more personalized interventions. These strategies can be offered to patients prospectively from the point at which they access the DMHI and may help identify subgroups of patients who are less likely to engage in treatment, based on characteristics they report at assessment, to facilitate a discussion about the appropriateness of the specific intervention.

## 6 Conclusion

This article proposes a person-centered model of patient complexity in which clinical and social factors accumulate and interact to shape uptake, engagement, and adherence to DMHIs. Integrating prior literature in other domains of healthcare, and complementary to existing models of DMHI engagement, the DiCuCoM emphasizes individual-level functions whereby complicating factors of workload, capacity, and treatment and illness burdens influence engagement. The focus on function facilitates a cohesive, generalizable framework with practical applicability to digital and other psychological interventions. The model also guides improvements and advances DMHI design and practice. Overall, the DiCuCoM is intended to stimulate innovations in research and practice that respect the clinical importance of workload-capacity imbalances and its effect on engagement equity. By deliberately considering the burden of treatment, DMHIs can evolve to mitigate systemic inequities, thereby supporting more equitable access to and engagement with mental health care across diverse user groups.

## Data Availability

The original contributions presented in the study are included in the article/supplementary material. Further inquiries can be directed to the corresponding author.
